# Identification of early invisible acute ischemic stroke in non-contrast computed tomography using two-stage deep-learning model

**DOI:** 10.7150/thno.74125

**Published:** 2022-07-18

**Authors:** Jun Lu, Yiran Zhou, Wenzhi Lv, Hongquan Zhu, Tian Tian, Su Yan, Yan Xie, Di Wu, Yuanhao Li, Yufei Liu, Luyue Gao, Wei Fan, Yan Nan, Shun Zhang, Xiaolong Peng, Guiling Zhang, Wenzhen Zhu

**Affiliations:** 1 Department of Radiology, Tongji Hospital, Tongji Medical College, Huazhong University of Science and Technology, Wuhan, China; 2 Department of CT & MRI, The First Affiliated Hospital, College of Medicine, Shihezi University, Shihezi, China; 3 Department of Artificial Intelligence, Julei Technology, Wuhan, China; 4 Athinoula A. Martinos Center for Biomedical Imaging, Department of Radiology, Massachusetts General Hospital, Harvard Medical School, Charlestown, MA, USA

**Keywords:** artificial intelligence, deep-learning, acute ischemic stroke, non-contrast computed tomography, diagnosis

## Abstract

**Rationale:** Although non-contrast computed tomography (NCCT) is the recommended examination for the suspected acute ischemic stroke (AIS), it cannot detect significant changes in the early infarction. We aimed to develop a deep-learning model to identify early invisible AIS in NCCT and evaluate its diagnostic performance and capacity for assisting radiologists in decision making.

**Methods**: In this multi-center, multi-manufacturer retrospective study, 1136 patients with suspected AIS but invisible lesions in NCCT were collected from two geographically distant institutions between May 2012 to May 2021. The AIS lesions were confirmed based on the follow-up diffusion-weighted imaging and clinical diagnosis. The deep-learning model was comprised of two deep convolutional neural networks to locate and classify. The performance of the model and radiologists was evaluated by the area under the receiver operator characteristic curve (AUC), sensitivity, specificity, and accuracy values with 95% confidence intervals. Delong's test was used to compare the AUC values, and a chi-squared test was used to evaluate the rate differences.

**Results:** 986 patients (728 AIS, median age, 55 years, interquartile range [IQR]: 47-65 years; 664 males) were assigned to the training and internal validation cohorts. 150 patients (74 AIS, median age, 63 years, IQR: 53-75 years; 100 males) were included as an external validation cohort. The AUCs of the model were 83.61% (sensitivity, 68.99%; specificity, 98.22%; and accuracy, 89.87%) and 76.32% (sensitivity, 62.99%; specificity, 89.65%; and accuracy, 88.61%) for the internal and external validation cohorts based on the slices. The AUC of the model was much higher than that of two experienced radiologists (65.52% and 59.48% in the internal validation cohort; 64.01% and 64.39% in external validation cohort; all *P* < 0.001). The accuracy of two radiologists increased from 62.00% and 58.67% to 92.00% and 84.67% when assisted by the model for patients in the external validation cohort.

**Conclusions**: This deep-learning model represents a breakthrough in solving the challenge that early invisible AIS lesions cannot be detected by NCCT. The model we developed in this study can screen early AIS and save more time. The radiologists assisted with the model can provide more effective guidance in making patients' treatment plan in clinic.

## Introduction

Stroke is the second leading cause of death and the top-ranked cause of disability-adjusted life-years in the 50-year and older groups 1,2, with ischemic stroke accounting for almost 80% 3. The major randomized placebo-controlled trials showed that the treatment is beneficial if administered early after stroke onset, decreasing in value over time 4. Early and accurate diagnosis is critical in acute ischemic stroke (AIS) to ensure that patients with fibrinolysis (< 4.5 hours) and mechanical thrombectomy (6-24 hours) receive treatment in the fastest possible onset-to-treatment time 5-7.

Time-efficient neuroimaging plays an important role in AIS diagnosis. Although MRI can reliably detect brain abnormalities in AIS 8, its clinical application is limited by contraindications, patient's incoordination, and high cost. Comparatively, non-contrast CT (NCCT) has the advantages of fast execution, effective exclusion of intracerebral hemorrhage, broad application in emergencies, and low cost. It is strongly recommended as the requisite examination for all patients with suspected AIS according to 2019 AHA/ASA guidelines 9. However, NCCT imaging hardly shows significant changes until 12-24 hours after the onset of stroke. The sensitivity of CT to detect AIS with more than 12 hours of onset time is only 16% and lowered to 12% within the first three hours 10. Additionally, even with abundant experience, radiologists may miss or misdiagnose when evaluating imaging data under emergency pressure 11. Hence, accurate and timely NCCT imaging diagnosis before treatment is a great challenge for radiologists.

Emerging artificial intelligence (AI) techniques, such as convolutional neural networks, hold promise and demonstrate efficiency and accuracy in performing imaging-based tasks 12-14. Using AI for image post-processing and interpretation in stroke can recognize in-depth information and reduce the differences between radiologists 15. The AI model has shown great performance in detecting large vessel occlusion through the cerebral artery hyperdense sign in NCCT, and automated Alberta Stroke Program Early CT Score (ASPECTS) rating with lesions in the middle cerebral artery area 16-19, but few studies discussed the invisible multi-size lesions distributed over the entire brain.

This study aimed to develop a new diagnosis model for identifying invisible and various AIS lesions in NCCT. We exploited the advantages of the two deep-learning models and a large sample of data to determine the location and the probability of disease risk and evaluated the model's diagnostic performance and ability to assist radiologists in making decisions.

## Materials and Methods

### Study population

**Figure 1:**
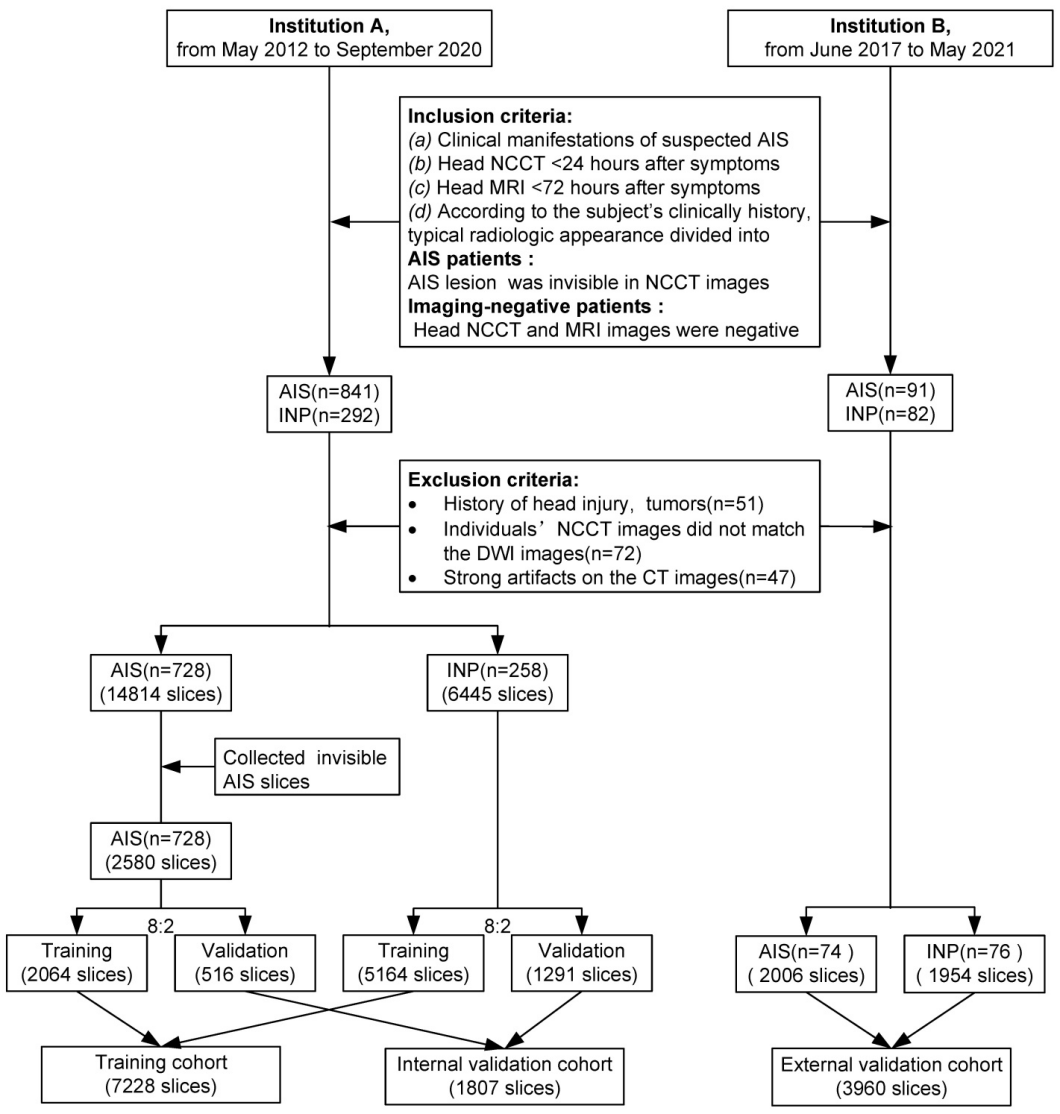
** Inclusion and exclusion workflow.** AIS = acute ischemic stroke, INP = imaging-negative patients

The data were retrospectively collected from two different institutions (Institution A: Tongji Hospital, including three districts, the Main Hospital Area, the Optical Valley Branch (Sino-German Friendship Hospital), and the Sino-French New City Branch; Institution B: The First Affiliated Hospital, School of Medicine, Shihezi University). Patients were included according to predefined criteria; any inconsistency was resolved by consensus. Inclusion criteria were *(a)* clinical manifestations with suspected AIS of limb numbness, limb weakness, dizziness, discomfort, etc., *(b)* head NCCT (slice thickness ≤ 5mm) within 24 hours after experiencing onset of stroke symptoms, *(c)* head MRI, including diffusion-weighted imaging (DWI) within 72 hours, and *(d)* based on the clinical history, manifestations, laboratory tests, treatment feedback, and typical radiologic appearance, the cohort was divided into AIS patients (AIS lesions in NCCT images were invisible, which was defined as AIS was not diagnosed by the junior radiologists) and imaging-negative patients(no AIS lesions and other abnormalities were in the images). Exclusion criteria were *(a)* a history of head injury or brain tumors, *(b)* head NCCT images did not match their DWI, and *(c)* strong artifacts on NCCT images. Slices of patients from institutions A were divided into training and internal validation groups by stratified random sampling at an 8:2 ratio. Patients from institutions B were assigned to the external validation cohorts (Figure 1).

### Image acquisition

All NCCT scans were obtained from one of the seven CT scanners (Optima 660, Discovery CT750 HD or Lightspeed VCT, GE Healthcare, America; Somatom Definition AS+, Siemens Healthineers, Germany; Brilliance iCT, Philips Healthcare, Netherlands; Aquilion ONE TSX 301A, Toshiba, Japan; uCT 780, United Imaging, China;) at institution A or one of the two CT scanners (Discovery CT750 HD and Lightspeed VCT, GE Healthcare, America) at institution B. CT protocols were 70-130 kVp, automatic tube current modulation(100-300 mA), 5 mm section thickness.

### Manual label of AIS NCCT images in the training cohort

The AIS NCCT images in the training cohort were labeled by two junior radiologists (SY and YX, < 3 years of experience). Using Pair software (RayShape Medical Technology, Shenzhen, China. http://www.aipair.com.cn/), a rectangular box was labeled at the AIS lesion in head NCCT images for detection. DWI images corresponding to the head CT images were juxtaposed slice-by-slice during the labeling process. The two junior radiologists participated in a training process until they had achieved 95% agreement in labeling the same 30 patients. After labeling, the intersection of union was greater than 0.8 between the random samples of the regions of interest delineated by the two radiologists (Figure S1).

### Preprocessing

The image contrast of all head NCCT images was standardized according to brain windows (window level: 30 HU, window width: 60 HU). Images were subjected to online image augmentation techniques such as horizontal flipping, cropping, and random rotation while training the models. The data augmentation strategy has been proven to help prevent network overfitting and memorization of the exact details of the training images.

### Deep-learning model and network architectures

**Figure 2:**
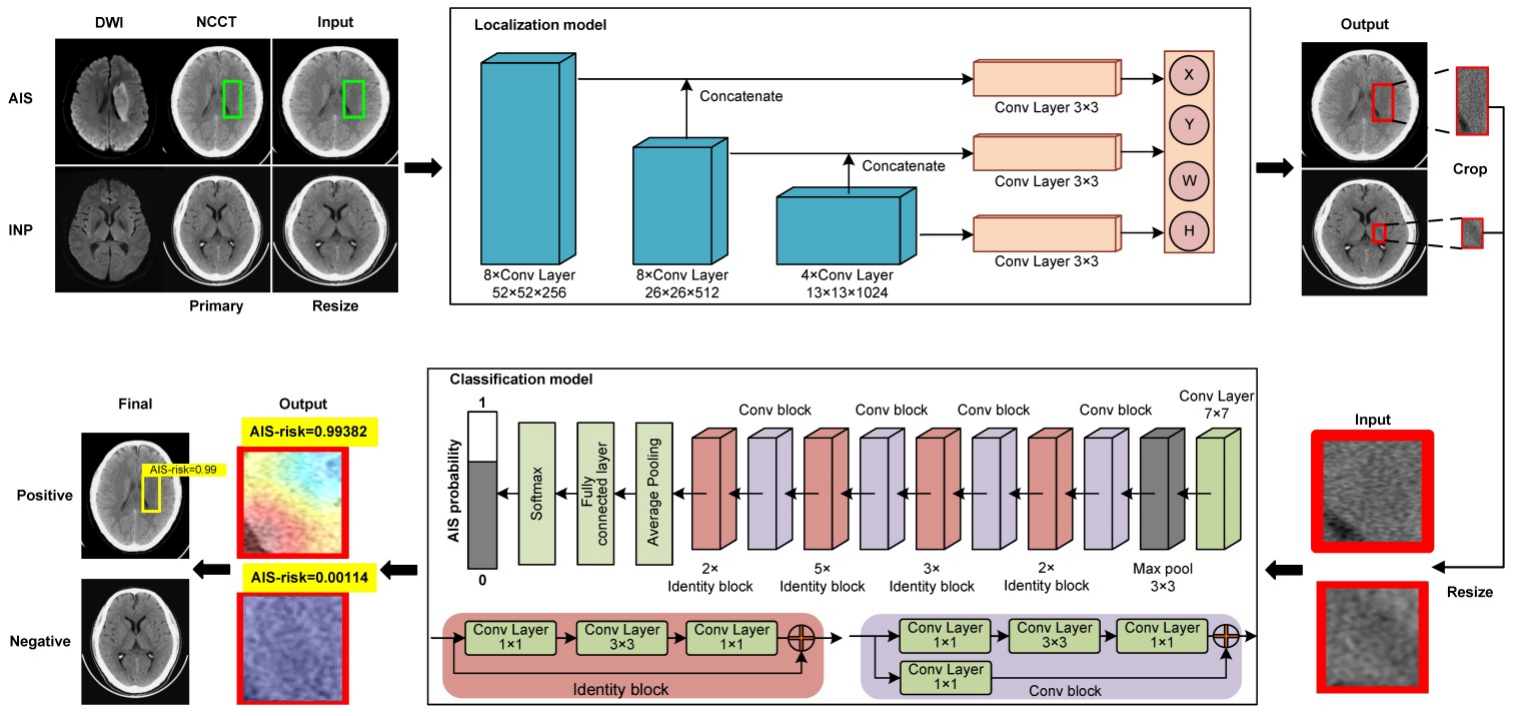
** Model training process.** The deep learning model is comprised of a localization model and a classification model. To train the localization model, the labeled AIS NCCT slices (green box) and negative NCCT slices were input, and the suspected regions were labeled and output (red box). The regions were cropped from the slice and normalized then input the classification model for training, output the diagnosis probabilities of AIS (AIS-risk) in these regions. The final results of this model are the lesions' location and probabilities of AIS (yellow box with AIS-risk). AIS = acute ischemic stroke, INP = imaging-negative patients.

We constructed a deep-learning model comprised of two deep convolutional neural networks (localization model and classification model) for diagnosing early invisible AIS in NCCT (Figure 2). The AIS localization model took an NCCT slice with an AIS label as the input and produced spatial coordinates as the output by the localization network. The classification model took the normalized AIS region in an NCCT slice and produced a final probability on whether it is negative or AIS by a classification network.

The localization model was based on YOLO v3, which could directly output the class probability and spatial coordinates. In the model training phase, the dataset was the AIS NCCT slices from the primary and internal validation cohorts. Before training, all NCCT slices were resized to the 416 × 416 dimensions, and intensities were normalized to (0, 1) range to balance computation cost and accuracy. The localization network was trained for 200 epochs with batch size at 8. The transfer learning was used to improve the performance, and the localization network was initialized by the pre-trained weight from the Microsoft Common Objects in the Context (MS-COCO) dataset. We employed the Adam optimizer with an initial learning rate of 0.0001, β1 at 0.9, β2 at 0.999. The learning rate decayed by a factor of 0.334 for every 5 epochs when there was no further improvement in the accuracy of the inter-validation set for 5 continuous epochs. Finally, the weight with the lowest validation loss was selected.

The AIS region was cropped from the slice using the trained localization network and normalized to the 64 × 64 dimensions. The classification network took the predictive AIS region as the input and output diagnosis probability of AIS, based on the 50-layer Residual Network (ResNet 50). During training, the weight of the network was initialized according to the weight from the pre-trained model on ImageNet. The SGD optimizer was employed with an initial learning rate of 0.005, momentum at 0.9, and weight decay at 0.0001. The learning rate decayed by a factor of 0.334 for every 20 epochs. The training epoch was 500 in total. To prevent overfitting, we used dropout, L2 regularization, and an early stopping strategy and finally selected the weight with lowest validation loss.

All procedures were conducted using Python (version 3.6.2), Keras (version 2.3.1), and Tensorflow (version 2.0.0) on NVIDIA GeForce 2080Ti graphical processing units.

### Evaluation

*Internal validation cohorts.* 516 AIS NCCT slices and 1291 negative NCCT slices in the internal validation cohorts were assessed by two experienced radiologists (YN and SZ, with 10 and 9 years of experience, respectively). The radiologists read images and signed the location and boundary of suspected AIS in NCCT slices without clinical diagnoses and MRI images of patients.

*External validation cohorts.* 74 AIS patients (2006 NCCT slices, 154 NCCT slices with AIS lesions, 1852 NCCT slices without lesions) and 76 imaging-negative patients (1954 negative NCCT slices) were read twice by two experienced radiologists. First, the radiologists read the images independently. Subsequently, they referred to the results of the deep-learning model and then decided whether to change the results of the first reading. The consistency between the regions of interest delineated by the radiologists and the output of the model was defined when the overlap was greater than 0.5.

### Statistical methods

Receiver operating characteristic (ROC) analysis was used to evaluate response prediction performance. The area under the ROC curve (AUC), accuracy, sensitivity, and specificity values with 95% confidence intervals (CIs) were reported for both the deep-learning model and radiologists. Delong's test was used to compare the AUCs. Pearson's chi-squared test, McNemar's test, or Fisher's exact test was used to evaluate the different rates. Mann-Whitney U test was used to compare quantitative variables. All statistical tests were two-sided, *P* < 0.05 was considered statistically significant. Statistical analyses and graphing were done on R version 3.6.1 (http://www.Rproject.org) and SPSS Statistics (version 22.0.0, IBM SPSS Statistics, Armonk, New York).

## Results

### Patients' images dataset and demographic characteristics

This study included 1306 patients (Figure 1); among them, 51 patients had a history of head injury or brain tumors (Institution A, 35), 72 NCCT images did not match their DWI (Institution A, 67), and 47 patients with strong artifacts on NCCT images (Institution A, 45) were excluded. The final number of patients included in this study was 1136. Of 986 patients from institution A (median age, 55 years; interquartile range [IQR]: 47-65 years; 664 males), 728 were diagnosed with AIS (569 males). NCCT was performed on these patients within a median of 8.03 hours from onset to scan (IQR: 3.5-18.69 hours), and the median baseline NIHSS was 4 (IQR: 2-7). From institution B, 150 patients (median age, 63 years, IQR: 53-75 years; 100 males) were included as the external validation group; NCCT was performed on 74 diagnosed with AIS (59 males) within a median of 9.5 hours from onset to scan (IQR: 5.75-16.07 hours). The median baseline NIHSS was 3 (IQR: 2-4.5). In 802 AIS patients, basal ganglia and corona radiata were most frequently affected, and most usually lesions diameter were 10-30 mm (Table 1).

### Performance of the deep-learning model

The deep-learning model performed well in diagnosing AIS lesions. The AUCs, sensitivities, specificities, and accuracies were 82.05% (95% CI: 81.01-83.08%), 66.28% (95% CI: 64.10-68.32%), 97.81% (95% CI: 97.41-98.20%), and 88.81% (95% CI: 88.06-89.53%), respectively, in training cohort and 83.61% (95% CI: 81.01-83.08%), 68.99% (95% CI: 65.12-73.06%), 98.22% (95% CI: 97.44-98.92%), and 89.87% (95% CI: 88.39-91.23%), respectively, in internal validation cohort (Table 2); these parameters in the external validation cohort were 76.32% (95% CI: 72.46-80.17 %), 62.99% (95% CI: 55.19-70.13%), 89.65% (95% CI: 88.68-90.57%), and 88.61% (95% CI: 87.58-89.58%), respectively (Table 3).

### Comparison between the deep-learning model and two radiologists in the internal validation cohort

**Figure 3:**
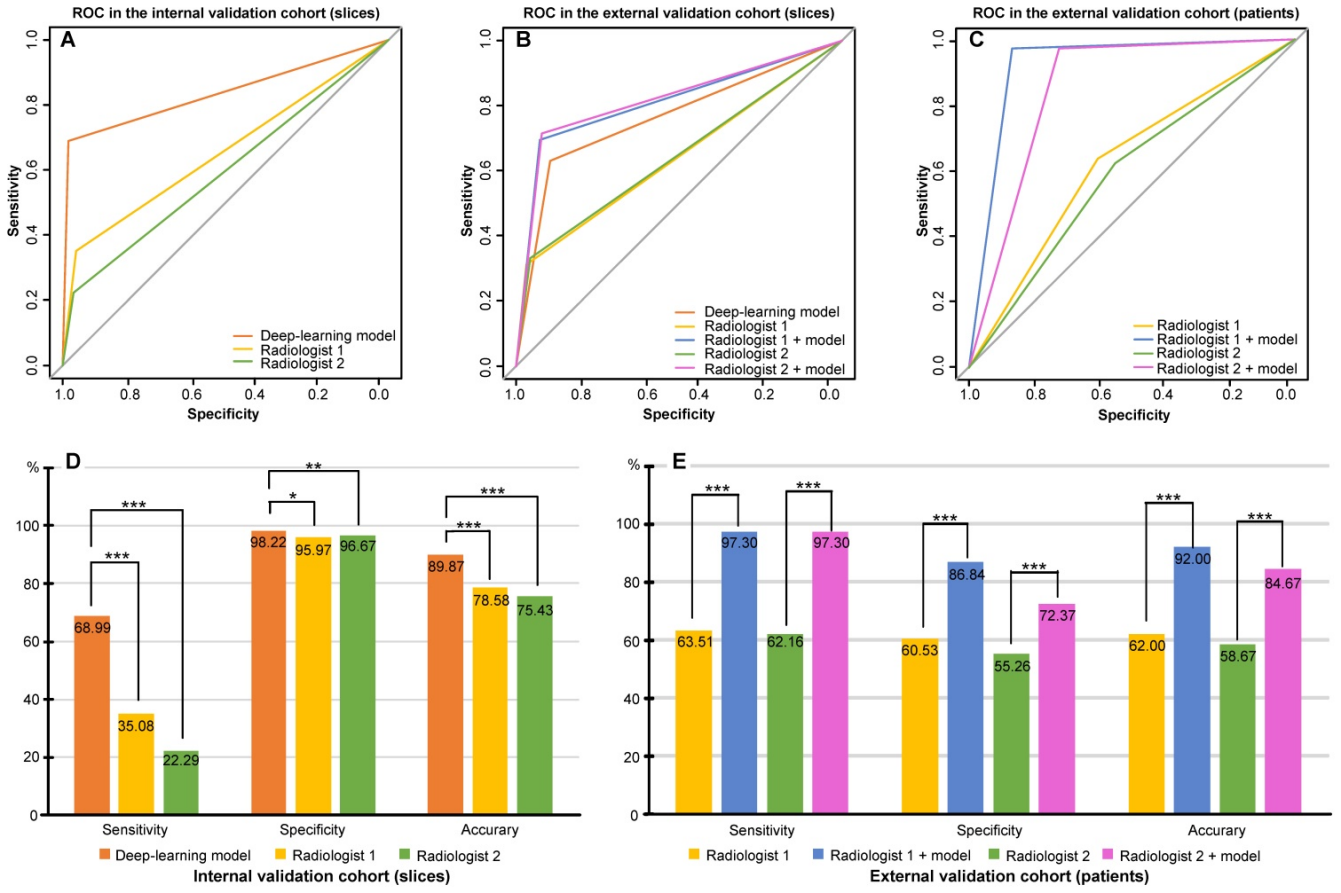
** Comparison of performance between the deep-learning model and experienced radiologists.** A. Receiver operating characteristic curve (ROC) of the deep-learning model and that from the two experienced radiologists for AIS detection based on slices in the internal validation cohort; B. ROC of the deep-learning model and that from the two experienced radiologists with and without the assistance of the model for AIS detection based on slices in the external validation cohort; C. ROC of each of the two experienced radiologists with and without the assistance of the model for AIS detection based on patients in the external validation cohort; D. Sensitivity, specificity, and accuracy of the deep-learning model and that from the two experienced radiologists for AIS detection in the internal validation cohort; E. Sensitivity, specificity, and accuracy of each of the two experienced radiologists with and without the assistance of the model for AIS detection based on patients in the external validation cohort.* 0.01 ≤ *P* < 0.05; **0.001 ≤ *P* < 0.01; *** *P* < 0.001.

The AUCs obtained by the radiologists 1 and 2 were 65.52% (95% CI: 63.40-67.65%) and 59.48% (95% CI: 57.62-61.34%). Their respective sensitivities, specificities, and accuracies were 35.08% (95% CI: 30.81-39.34%), 95.97% (95% CI: 94.89-96.98%), and 78.58% (95% CI: 76.62-80.45%), respectively, for radiologist 1 and 22.29% (95% CI: 18.60-25.78%), 96.67% (95% CI: 95.66-97.60%), and 75.43% (95% CI: 73.38-77.40%), respectively, for radiologist 2 (Table 3). The AUC of the deep-learning model was much higher than that from the two radiologists in the internal validation cohort (*P* < 0.001) (Figure 3A) as was the case for accuracy of the model (Figure 3D, Table S1).

### Comparisons between two radiologists without and with the assistance of the deep-learning model in the external validation cohort

For radiologist 1, 64.01% AUC (95% CI: 60.33-67.69%), 31.17% sensitivity (95% CI: 24.03-38.33 %), 96.85% specificity (95% CI: 96.30-97.40%), and 94.29% accuracy (95% CI: 93.52-94.50%) were reported without the model, while with the assistance of the model, the values were 81.15% AUC (95% CI: 77.48-84.83%), 69.48% sensitivity (95% CI: 62.34-76.62%), 92.83% specificity (95% CI: 91.96-93.59%), and 91.92% accuracy (95% CI: 91.03-92.75 %). Similarly, for radiologist 2, the values were 64.39% AUC (95% CI: 60.65-68.13%), 33.12% sensitivity (95% CI: 25.97-40.91%), 95.66% specificity (95% CI: 95.01-96.32%), and 93.23% accuracy (95% CI: 92.41-94.00%) without the model and improved to 81.83% AUC(95% CI: 78.22-85.43%), 71.43% sensitivity (95% CI: 64.29-78.57%), 92.22% specificity (95% CI: 91.33-93.09%), and 91.41% accuracy (95% CI: 90.50-92.27%) with the model (Table 3). Thus, both radiologists achieved greater predictive performances with the help of the model than without the model (*P* < 0.001). Based on the AUCs, the model exhibited a higher predictive performance than the radiologists (*P* < 0.001), but a lower performance compared to radiologists with the assistance of the model (*P* < 0.001) (Figure 3B).

**Figure 4:**
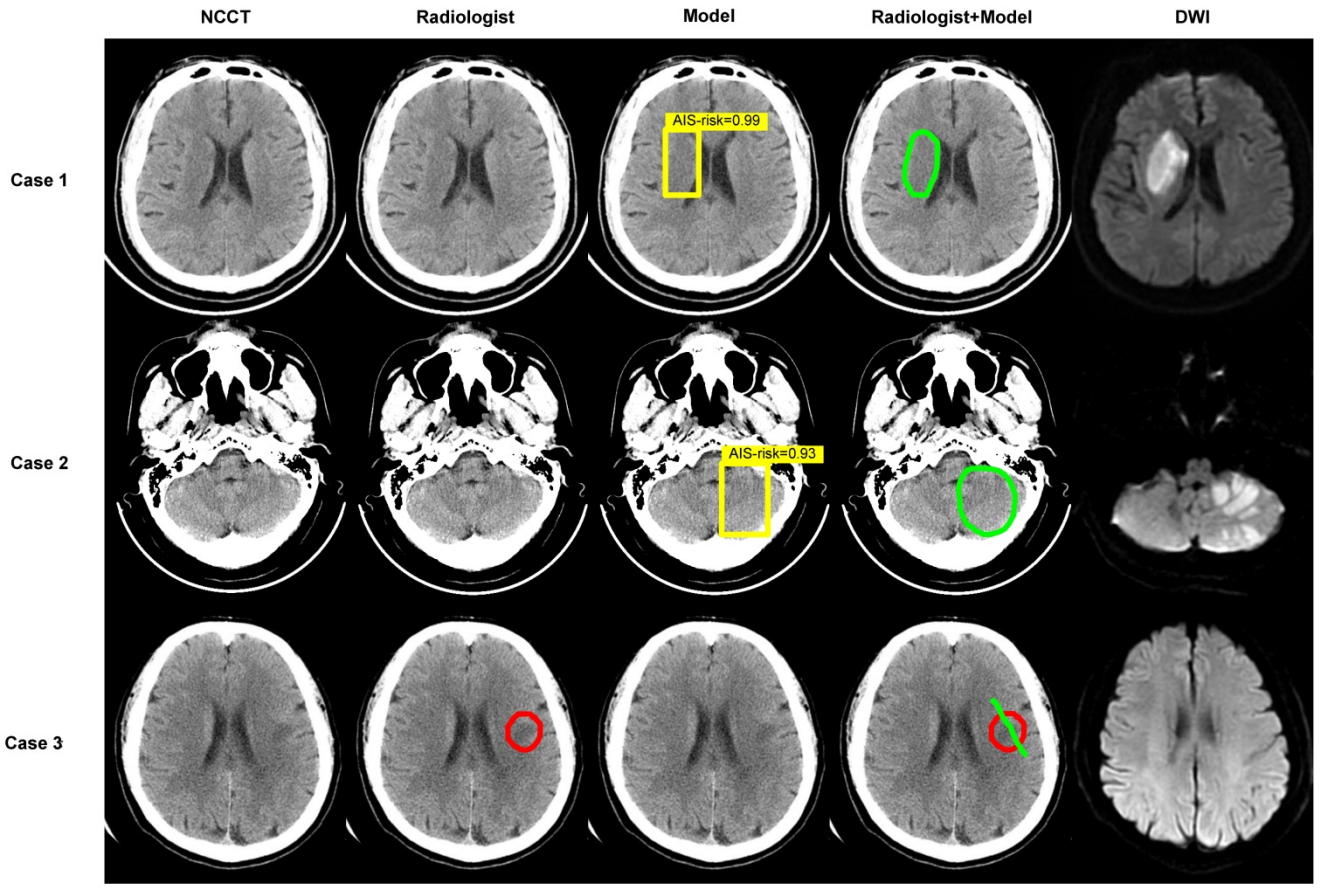
** Examples. Images show the results of two experienced radiologists without and with the assistance of the deep-learning model for diagnosing.** The first two cases are AIS patients and the last was imaging-negative patient. Within each case, left column shows a NCCT image, second column shows the result of radiologist read, middle column shows result of model diagnose (the yellow box represents the location of AIS and the probabilities calculated by the model in this region are 0.99, 0.93), fourth column shows the result of “radiologist + model” (the green circle or line), and right column shows corresponding DWI.

We performed analyses by converting the slices into patients. In accordance with the rules, when at least one AIS NCCT slice was detected, the AIS patient was recorded as true positive. In patients with negative imaging, the absence of lesions in all slices was considered true negative. Table 3 shows an AUC of 62.02% (95% CI: 54.20-69.84%), 63.51% sensitivity (95% CI: 52.67-74.32%), 60.53% specificity (95% CI: 48.68-71.05%), and 62.00% accuracy (95% CI: 53.72-69.79%) without the model and 92.07% AUC (95% CI: 87.82-96.32%), 97.30% sensitivity (95% CI: 93.24-100.00%), 86.84% specificity (95% CI: 78.95-93.42 %), and 92.00% accuracy (95% CI: 86.44-95.08%) with the model for radiologist 1. For radiologist 2, the reported values were 58.71% AUC (95% CI: 50.80-66.62%), 62.16% sensitivity (95% CI: 51.35-72.97%), 55.26% specificity (95% CI: 44.74-67.11%), and 58.67% accuracy (95% CI: 50.35-66.64%) without the model and 84.83% AUC (95% CI: 79.44-90.22%), 97.30% sensitivity (95% CI: 93.24-100%), 72.37% specificity (95% CI: 61.84-81.58%), and 84.67% accuracy (95% CI: 77.89-90.02%) with the model. The AUCs achieved by radiologists with the assistance of the model showed greater predictive performance than without the model (*P* < 0.001) (Figure 3C). Besides, sensitivity, specificity, and accuracy by radiologists with the assistance of the model were higher than those without the model (*P* < 0.001) (Figure 3E, Table S2). Of 150 patients, the number diagnosed correctly increased by 45 (radiologist 1) and 39 (radiologist 2) while using the deep-learning model. Figure 4 displays three examples without and with the assistance of the model reported by radiologists.

### Comparisons between different manufacturers

In the internal validation cohort, NCCT was acquired by using GE Healthcare in 394 patients (236 with AIS, 360 slices), SIEMENS in 55 patients (38 with AIS, 60 slices), Toshiba in 62 patients (22 with AIS, 31 slices), United Imaging in 62 patients (44 with AIS, 64 slices), and Philips in 1 patient (with AIS, 1 slice, not included in the comparison). The AUCs were 84.37% (95% CI: 81.97-86.77%), 80.34% (95% CI: 74.06-86.62%), 82.05% (95% CI: 73.48-90.62%), and 82.31% (95% CI: 76.37-88.26%) for GE Healthcare, SIEMENS, Toshiba, and United Imaging, respectively, without significant differences (for each pairwise comparison, *P* > 0.5). Thus, the comparison of model sensitivities and specificities across different manufacturers was not statistically significant (*P* > 0.1) (Table S3 and Figure S2).

## Discussion

In this study, we developed a two-stage deep-learning model to diagnose the early invisible AIS lesions of diverse sizes distributed in all brain regions in NCCT and verified the model using a multi-center, multi-manufacturer platform. The model achieved satisfactory performance in diagnosing early invisible AIS in the training, internal and external validation cohorts with AUCs of 82.05%, 83.61% and 76.32%, respectively. The model outperformed two experienced radiologists in the internal and external validation cohorts (AUC: 83.61% vs 65.52% and 59.48%,* P* < 0.001; 76.32% vs 64.01% and 64.39%, *P* < 0.001). By incorporating the model in the analyses, the accuracy of two radiologists improved from 62.02% and 58.71% to 92.07% and 84.83%, respectively, based on patients in the external validation cohorts.

Of the 1136 eligible patients enrolled in our study, 802 patients had AIS lesions distributed in all supra- and infra-tentorial brain regions, including pons, and could mistakenly be considered by radiologists as stroke lesions in clinical diagnosis. The maximum cross-sectional diameter of the lesions ranged between 3-158 mm, including lacunar infarction and large infarct. The inclusion of patients with entirely negative NCCT presentation, increasing the difficulty of model detection, showed the excellent specificity of the model. Qiu and colleagues combined manually defined and deep-learning features to detect and quantify infarcts on baseline NCCT images, but their study only included lesions in the M1 segment area of the middle cerebral artery 20. Similarly, Kniep et al. studied only the posterior circulation stroke, which was not consistent with the diversity seen in the clinic 21.

Our study was a multi-center and multi-manufacturer study. Institution A had three hospital districts, and the good performance of the model in the internal validation cohort could be used primarily to prove its robustness. Institution B was several thousand miles away from institution A with different demographic compositions and scanning parameters. Although there were differences in some clinical features between the two institutions, the results from the external validation set showed that the model could still make effective predictions and demonstrate good generalizability and robustness. Also, the model showed comparable diagnostic performance using instruments from different manufacturers.

Previously, a deep-learning model was used by Nishio et al. to detect AIS lesions 22. In another study, Davis developed a method based on the symmetry of cerebral hemispheres for detecting the early stages of ischemic stroke 23. However, the robustness of the methods was not verified. The model presented here displayed high generalizability and robustness. Our study considered a large number of patients, the diversity of lesion distribution and size, and the potential for easy misdiagnosis and underdiagnosis. The multi-center and multi-manufacturer application aspects made our model building more consistent with clinical reality and made it easier to apply it in clinical settings.

Our study benefited from the rapid development of deep-learning techniques building on the innovative advances. The localization model was built on YOLO v3, which divides the entire image into multiple regions and predicts bounding boxes and probabilities for each region 24. ResNet was used for classification, achieving accuracy from a substantially increased depth 25. We combined the advantages of localization and classification networks, which could inform the region of the lesions and the degree of disease risk. The localization network trained the model by learning the labeled AIS NCCT slices but could not learn the negative NCCT slices. Although using NCCT slices could minimize the possibility of false information in the images, the trained localization network had high sensitivity in finding AIS with high a false-positive rate and did not theoretically predict negative NCCT images. We introduced the classification network as the second stage model and removed the category loss function to reduce the false-positive rate. Therefore, our two-stage model could automatically locate and classify AIS with high predictive performance.

As for the ROC curves, this model significantly outperformed two experienced radiologists. Significantly, the radiologists' diagnostic accuracy of AIS improved with the assistance of the model. Wu et al. also proposed a model for identifying invisible ischemic strokes in NCCT 26 and Pan et al. used ResNet to detect infarct core on NCCT with an accuracy of 75.9% 27. Their study did not compare the performance of radiologists to determine the effectiveness of model assistance. We have demonstrated that the deep-learning model we developed could significantly improve the diagnostic performance of radiologists. Artificial intelligence has the potential to fully assess the heterogeneity known to be associated with underlying biology. The radiologists can advise clinicians in deciding on treatment strategies and management by combining the results of the model with clinical information.

This study has some limitations. Although the detailed clinical information may improve the performance, we did not incorporate it in the model for two reasons. First, from an objective clinical perspective, many emergency patients may not be able to provide enough clinical information, and second, similar clinical manifestations of normal patients and AIS may easily confuse the judgment of the doctors. Our premise for building this model was that the AI diagnostic system could still be efficacious even in cases of incomplete clinical information. Another limitation is the time interval between CT and MRI. CT was acquired within 24 hours of the onset of symptoms, while MRI (including DWI) was done within 72 hours, which potentially makes the lesion in MRI not fully representative of the actual lesion in CT. Considering this, we did not outline the boundary of the lesion exactly according to MRI in CT but used a box to visualize the location and size of the lesion. For AIS, which is invisible to the naked eye of the observer in CT, it is important to indicate the lesion location and size using the model. Besides, the sensitivity for AIS detection was relatively low as the AIS lesions were invisible in NCCT. Also, the AIS NCCT slices accounted for a small proportion of the AIS patients' NCCT, leading to a large difference in the number of positive and negative slices. In future studies, we will focus on the ratio of the number of positive to negative slices to minimize the difference. Most importantly, we will increase the sample size by enrolling patients from multiple centers.

## Conclusions

We developed a deep-learning model to diagnose early invisible AIS lesions in NCCT using two-stage deep convolutional neural networks with better robustness. The model has a much higher diagnostic power than experienced radiologists and can significantly improve the sensitivity and accuracy of the diagnosis. With the help of the model, radiologists can make better decisions and select more appropriate treatment methods.

## Supplementary Material

Supplementary figures and tables.

## Figures and Tables

**Table 1:** Patient demographic data

	Institution A (n = 986)	Institution B (n = 150)	*P*
Subjects characters*			
Diagnosis (n)			< 0.0001^¥^
AIS (slices)	728 (2580)	74 (2006)	
INP (slices)	258 (6445)	76 (1954)	
Age, years, median [IQR]	55 [47-65]	63 [53-75]	< 0.0001^§^
Gender (n)			0.8690^¥^
Male	664	100	
Female	322	50	
Clinical information^※^			
TSS, hours, median [IQR]	8.03 [3.50-18.69]	9.50 [5.75-16.07]	0.7020^§^
NIHSS, median [IQR]	4.00 [2.00-7.00]	3.00 [2.00-4.50]	0.0090^§^
Lesions features^※^			
Number (n)			0.3010^¥^
Single	509	56	
Multiple	219	18	
Location (n)			0.0050^¥^
Left	341	32	
Right	331	28	
Bilateral	56	14	
Size (mm)			< 0.0001^¥^
0-10	257	42	
10-30	522	50	
30-50	151	8	
50-100	102	3	
> 100	32	0	
Area (n)			< 0.0001^¶^
Frontal lobe	176	10	
Parietal lobe	162	3	
Temporal lobe	188	8	
Occipital lobe	70	4	
Insular lobe	105	3	
Basal ganglia	273	35	
Corona radiata	247	41	
Centrum semiovale	88	6	
Pons	54	6	
Mesencephalon	50	7	
Cerebellum	45	6	
Cerebral peduncle	8	5	
Thalamus	64	4	
Corpus callosum	19	2	
Hippocampus	12	2	
Periventricular	20	2	

AIS = acute ischemic stroke, NIP = imaging-negative patients, TTS = time from onset to scan, NIHSS = National Institute of Health stroke scale, IQR = interquartile range * For the entire cohort of 1136 subjects. ※ For the lesions in 802 AIS patients. ¥: The Pearson's chi-squared test was performed. §: The Mann-Whitney U test was performed. ¶: The Fisher's exact test was performed.

**Table 2:** Performance of deep learning model and two experienced radiologists in training and internal validation cohort

	Results (n)				Test performance (%)				
	TP	TN	FP	FN	AUC [95%CI]	Sensitivity [95%CI]	Specificity [95%CI]	Accuracy [95%CI]	*P* ^†^
Training	1368	5051	113	696	82.05 [81.01-83.08]	66.28 (1368/2064) [64.10-68.32]	97.81 (5051/5164) [97.41-98.20]	88.81 (6419/7228) [88.06-89.53]	
Internal Validation									
Deep-learning model	356	1268	23	160	83.61 [81.58-85.64]	68.99 (356/516) [65.12-73.06]	98.22 (1268/1291) [97.44-98.92]	89.87 (1624/1807) [88.39-91.23]	
Radiologist 1	181	1239	52	335	65.52 [63.40-67.65]	35.08 (181/516) [30.81-39.34]	95.97 (1239/1291) [94.89-96.98]	78.58 (1420/1807) [76.62-80.45]	< 0.0001
Radiologist 2	115	1248	43	401	59.48 [57.62-61.34]	22.29 (115/516) [18.60-25.78]	96.67 (1248/1291) [95.66-97.60]	75.43 (1363/1807) [73.38-77.40]	< 0.0001

TP = true positive, TN = true negative, FP = false positive, FN = false negative †: compare between radiologists and deep learning model. Delong's test was used to compare the AUCs.

**Table 3:** Performance of the deep learning model and two experienced radiologists in external validation cohort

	Results (n)				Test performance (%)					
	TP	TN	FP	FN	AUC [95%CI]	Sensitivity [95%CI]	Specificity [95%CI]	Accuracy [95%CI]	*P* ^†^	*P* ^$^
Based on slices										
Deep-learning model	97	3412	394	57	76.32 [72.46-80.17]	62.99 (97/154) [55.19-70.13]	89.65 (3412/3806) [88.68-90.57]	88.61 (3509/3960) [87.58-89.58]		
Radiologist 1	48	3686	120	106	64.01 [60.33-67.69]	31.17 (48/154) [24.03-38.33]	96.85 (3686/3806) [96.30-97.40]	94.29 (3734/3960) [93.52-94.50]	< 0.0001	< 0.0001
Radiologist 1 **+** model	107	3533	273	47	81.15 [77.48-84.83]	69.48 (107/154) [62.34-76.62]	92.83 (3533/3806) [91.96-93.59]	91.92 (3640/3960) [91.03-92.75]	< 0.0001	
Radiologist 2	51	3641	165	103	64.39 [60.65-68.13]	33.12 (51/154) [25.97-40.91]	95.66 (3641/3806) [95.01-96.32]	93.23 (3692/3960) [92.41-94.00]	< 0.0001	< 0.0001
Radiologist 2 **+** model	110	3510	296	44	81.83 [78.22-85.43]	71.43 (110/154) [64.29-78.57]	92.22 (3510/3806) [91.33-93.09]	91.41 (3620/3960) [90.50-92.27]	< 0.0001	
Based on patients										
Radiologist 1	14	46	30	60	39.72 [32.60-46.85]	63.51 (47/74) [52.67-74.32]	60.53 (46/76) [48.68-71.05]	62.00 (93/150) [53.72-69.79]		< 0.0001
Radiologist 1 **+** model	40	66	10	34	70.45 [63.57-77.33]	97.30 (72/74) [93.24-100.00]	86.84 (66/76) [78.95-94.74]	92.00 (138/150) [86.44-95.80]		
Radiologist 2	16	42	34	58	38.44 [31.10-45.79]	62.16 (46/74) [51.35-72.97]	55.26 (42/76) [43.42-67.11]	58.67 (88/150) [50.35-66.64]		< 0.0001
Radiologist 2 + model	41	55	21	33	63.89 [56.26-71.51]	97.30 (72/74) [93.24-100.00]	72.37 (55/76) [61.84-82.89]	84.67 (127/150) [77.89-90.02]		

TP = true positive, TN = true negative, FP = false positive, FN = false negative †: compare between radiologists and deep learning model. $: compare between the radiologists and radiologists +model. Delong's test was used to compare the AUCs.

## Abbreviations

AI: artificial intelligence
AIS: acute ischemic stroke
ASPECTS: Alberta stroke program early computed tomography score
AUC: area under the curve
CI: confidence interval
CT: computed tomography
DWI: diffusion weighted imaging
MRI: magnetic resonance imaging
NCCT: non-contrast computed tomography
ROC: receiver operator characteristic
