# Supplementary material for: Identification of early invisible acute ischemic stroke in non-contrast computed tomography using two-stage deep-learning model
Source: Theranostics. 2022 Jul 18;12(12):5564–73. doi: 10.7150/thno.74125 (PMC9330528; doi:10.7150/thno.74125)
Supplement: Supplementary file 1 — Supplementary figures and tables. [file thnov12p5564s1.pdf]

Supplementary Material

Table S1: Comparisons of the deep-learning model and two experienced radiologists based on slices in the internal validation cohort

	Test performance (%)		
	Sensitivity [95%CI]	Specificity [95%CI]	Accuracy [95%CI]
**Deep-learning model**	68.99 (356/516) [65.12-73.06]	98.22 (1268/1291) [97.44-98.92]	89.87 (1624/1807) [88.39-91.23]
**Radiologist 1**	35.08 (181/516) [30.81-39.34]	95.97 (1239/1291) [94.89-96.98]	78.58 (1420/1807) [76.62-80.45]
χ^2	130.3191	11.5206	135.1169
$P^{\dagger}$	< 0.0001	0.0009	< 0.0001
**Radiologist 2**	22.29 (115/516) [18.60-25.78]	96.67 (1248/1291) [95.66-97.60]	75.43 (1363/1807) [73.38-77.40]
χ^2	203.793	6.0606	194.0769
$P^{\dagger}$	< 0.0001	0.0187	< 0.0001

†: compare between radiologists and deep learning model.

The McNemar's test was performed.

Table S2: Comparisons of the two experienced radiologists without and with the model based on patients in the external validation cohort

	Test performance (%)		
	Sensitivity [95%CI]	Specificity [95%CI]	Accuracy [95%CI]
**Radiologist 1**	63.51 (47/74) [52.67-74.32]	60.53 (46/76) [48.68-71.05]	62.00 (93/150) [53.72-69.79]
**Radiologist 1 + model**	97.30 (72/74) [93.24-100.00]	86.84 (66/76) [78.95-94.74]	92.00 (138/150) [86.44-95.80]
χ^2	23.04	18.05	45
$P^{\$}$	< 0.0001	< 0.0001	< 0.0001
**Radiologist 2**	62.16 (46/74) [51.35-72.97]	55.26 (42/76) [43.42-67.11]	58.67 (88/150) [50.35-66.64]
**Radiologist 2 + model**	97.30 (72/74) [93.24-100.00]	72.37 (55/76) [61.84-82.89]	84.67 (127/150) [77.89-90.02]
χ^2	24.04	11.08	39
$P^{\$}$	< 0.0001	0.0009	< 0.0001

$: compare between the radiologists and radiologists + model
The McNemar's test was performed.

Table S3: Comparisons of deep-learning model with different manufacturers based on slice in the internal validation cohort

	Results (n)				Test performance (%)			
	TP	TN	FP	FN	AUC [95%CI]	Sensitivity [95%CI]	Specificity [95%CI]	Accuracy [95%CI]
**GE Healthcare**	256	825	20	104	84.37 [81.97-86.77]	71.11 (256/360) [66.39-75.83]	97.63 (825/845) [96.57-98.58]	89.71 (1081/1205) [87.85-91.37]
**SIEMENS**	37	100	1	23	80.34 [74.06-86.62]	61.67 (37/60) [50.00-73.33]	99.01 (100/101) [96.04-100.00]	85.09 (137/161) [78.64-90.21]
**Toshiba**	20	239	1	11	82.05 [73.48-90.62]	64.52 (20/31) [48.39-80.65]	99.58 (239/240) [98.75-100.00]	95.57 (259/271) [92.39-97.69]
**United Imaging**	42	99	1	22	82.31 [76.37-88.26]	65.63 (42/64) [54.69-76.56]	99.00 (99/100) [97.00-100.00]	85.98 (141/164) [79.70-90.90]
χ^2						2.886	4.901	16.433
$P^{‡}$					> 0.5	0.410[¥]	0.2020[¶]	0.0010[¥]

TP = true positive, TN = true negative, FP = false positive, FN = false negative
‡: compare between different manufacturers
Delong’s test was used to compare the AUCs.
¥: The Pearson’s chi-squared test was performed.
¶: The Fisher's exact test was performed.

Table S4: Comparison of YOLO v3 only and combined YOLO v3+ResNet results based on slice

	Results (n)				$P^{£}$
	TP	TN	FP	FN	
**Training**					
**YOLO v3**	1437	3838	1326	627	< 0.0001
**YOLO v3 + ResNet**	1368	5051	113	696	
**Internal Validation**					
**YOLO v3**	370	936	355	146	< 0.0001
**YOLO v3 + ResNet**	356	1268	23	160	

TP = true positive, TN = true negative, FP = false positive, FN = false negative
£: Compare between YOLO v3 and YOLO v3+ResNet
The McNemar's test was performed.

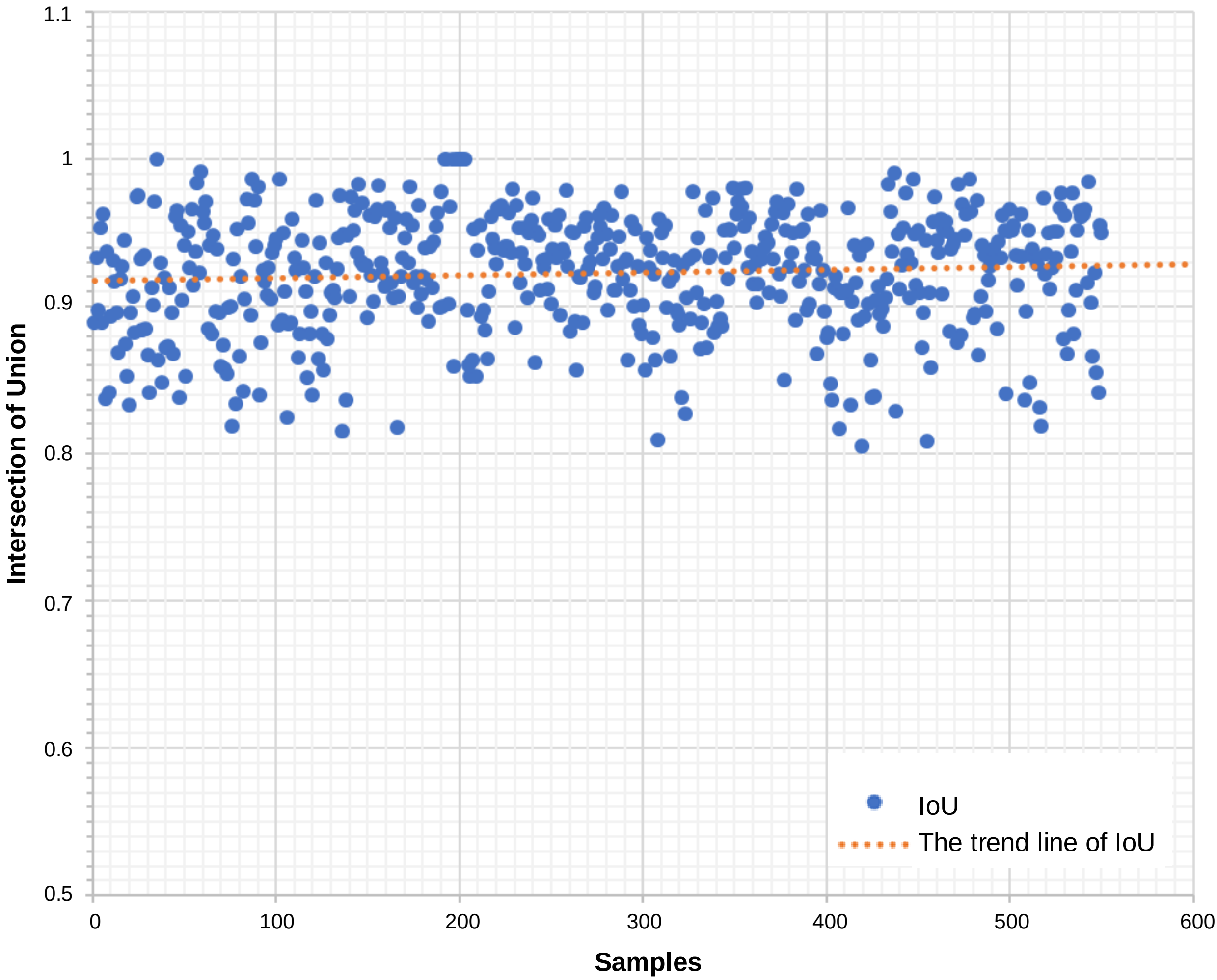

Figure S1. The IoU distribution of the random sample of the results labeled by the two radiologists. After labeling, the random sampling of the results labeled by the two radiologists were performed, and the intersection of union (IoU) were greater than 0.8.

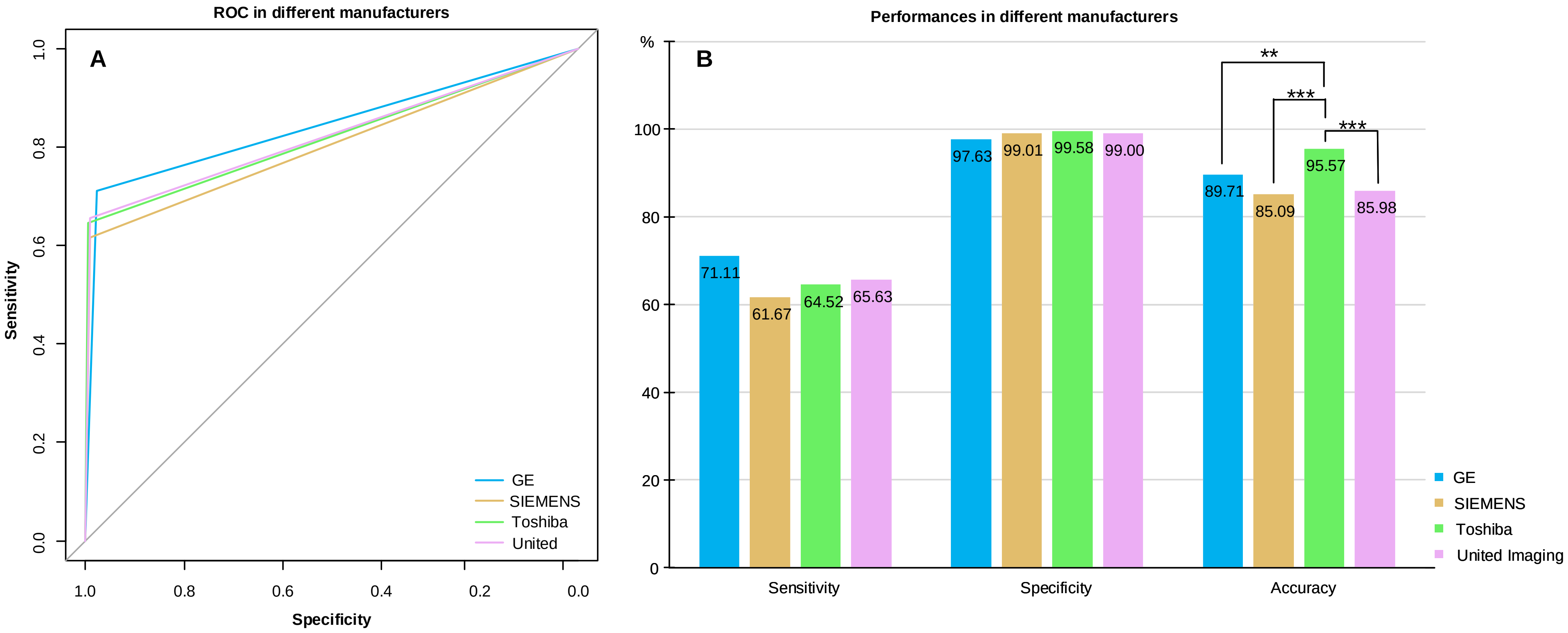

Figure S2. Comparisons of deep-learning model with different manufacturers. A. Receiver operating characteristic curve (ROC) of the deep-learning model with four different manufacturers based on slices in the internal validation cohort; **B.** Sensitivity, specificity, and accuracy of the deep-learning model with four different manufacturers for AIS detection in the internal validation cohort. The accuracy of 95.57% of Toshiba was higher than those of GE (89.71%, $P = 0.003$), SIEMENS (85.09%, $P < 0.001$), and United Imaging (85.98%, $P < 0.001$). The Fisher's exact test was performed. * $0.01 \leq P < 0.05$; **$0.001 \leq P < 0.01$; *** $P < 0.001$.
